# Suicide Ideation and Depression Quality of Life Ratings in a Reservation-Based Community of Native American Youths and Young Adults

**DOI:** 10.1007/s10597-021-00883-w

**Published:** 2021-08-29

**Authors:** Y. N. Alfonso, D. Bishai, J. D. Ivanich, V. M. O’Keefe, J. Usher, L. R. Aldridge, E. E. Haroz, N. Goklish, A. Barlow, M. Cwik

**Affiliations:** 1 Department of International Health, Johns Hopkins Bloomberg School of Public Health, 615 N. Wolfe Street, Baltimore, MD 21205 USA; 2 Department of Population, Family and Reproductive Health, Johns Hopkins Bloomberg School of Public Health, 615 N. Wolfe Street, Baltimore, MD 21205 USA; 3 Department of Community and Behavioral Health, Colorado School of Public Health, 13055 East 17th Avenue, Aurora, CO 80045 USA

**Keywords:** Quality-of-life, Suicide, Ideation, Depression, Adolescence, American Indian

## Abstract

Suicide among adolescents is a significant public health concern in the U.S., especially within American Indian and Alaska Native (AIAN) communities. Lack of quality of life (QoL) estimates for both suicide ideation and depression specific to the AIAN population hinders the ability to compare interventions in cost-effectiveness analysis. We surveyed 200 AI youth and young adults from the Fort Apache Indian Reservation to estimate utility weights for experiencing suicide ideation and depression. Our results indicate that, on a scale of 0–100, with higher scores indicating better health, the general community rates both suicide ideation and depression at 15.8 and 25.1, respectively. These weights are statistically significantly different and lower than for other cultures. Culturally specific QoL values will allow the comparison and identification of the most effective and feasible interventions to reduce the suicide burden among tribal communities.

**Supplementary Information:**

The online version contains supplementary material available at 10.1007/s10597-021-00883-w.

## Introduction

Suicide is the second leading cause of death among youth and young adults 10–24 years of age in the U.S. and poses an important challenge for public health (Heron, 2018). Although there are differences by tribal nation, American Indian and Alaska Native (AIAN) youth as a group bear a disproportionate burden of suicide, with a mortality rate 50% greater than their white peers (Herne et al., 2014). Major risk factors for suicide among AIAN include mental health problems such as depression, trauma, substance use, impulsivity, self-injury, low self-esteem, and hopelessness (M. Cwik et al., 2015). Public health efforts are underway to prevent suicide by targeting these risk factors and promoting resilience among AIAN youth.

Given the limited resources for public health efforts in Native communities, researchers must evaluate and consider both the clinical significance and cost-effectiveness of suicide prevention and treatment interventions. Therefore, cost-utility analyses (CUAs) are warranted to inform which interventions are most effective, cost-effective, and feasible. CUAs rely on utility weights to measure the quality of life during time spent in various health states. The QOL weights permit one to convert changes in time spent with a specific clinical health state to quality-adjusted life-years (QALYs) which can be compared across clinical conditions and interventions. A QALY is a population health measure that encompasses both changes to morbidity and mortality in a single metric. The utility weight is the key parameter needed to estimate the morbidity component of QALYs. Weights range on a scale of 0–1.0, where zero is the worst health imaginable (e.g. death) and one is the best health imaginable (e.g. a full and healthy life) (Drummond et al., 2005; Gold et al., 2002).

It is important to measure utility weights for both depression and suicide given that depressive symptoms and/or a diagnosis of major depressive disorder are strong risk factors for suicide ideation, attempts, and deaths (Bachmann, 2018; Ribeiro et al., 2018). Further, weights differ between communities because individual preferences for different health states and what is worse or better is influenced by the unique context in which an individual lives and their experiences. As such, it is vital to gain local community quality of life ratings pertaining to depression and suicide ideation. Utility weights have been well established for various states of depression and attempted suicide or suicide death for the general global population (Salomon et al., 2016). However, a search of the literature yields few studies estimating weights of suicidal ideation and none to date estimating depression or suicide ideation weights among AIAN populations (Bustamante Madsen et al., 2018; Kwon et al., 2018; Mohiuddin & Payne, 2014; Sonntag et al., 2013; Tufts Medical Center, 2013).

Goldney and colleagues (2001) estimated utility weights for suicide ideation among a nationally representative sample of Australians at 0.45, and van Skijker and colleagues using a panel of Dutch medical practitioners, estimated utility weights for suicide ideation in the Dutch population at 0.36 (Goldney et al., 2001; van Spijker et al., 2011). These weights indicate that among these populations, one year spent experiencing suicide ideation is equivalent to 0.45 or 0.36 QALYs, respectively. Pils and colleagues (2013) built on the methodology of the van Skijker and colleagues to develop weights for the Belgium population burdened with different suicide-related outcomes, ranging from suicide ideation to re-attempt, at 0.78–0.56) (Pil et al., 2013). Other research has estimated the utility weight of suicide nested within a larger health state, such as within complicated bereavement, ranging between 0.54–0.87 for control groups and 0.55–0.93 for intervention groups (Comans et al., 2013).

For depression, a recent systematic review found seven studies from the U.S., U.K., and Netherlands reporting weights for children and youth 12–19 years old with depression or being treated for depression at 0.57 with the visual analog scale instrument, 0.89 with the EuroQol instrument and 0.57 with other non-preference based measures (Kwon et al., 2018). Weights higher than 0.57 pertained to groups with treatment or who are depression-free. Other systematic reviews of utility weights of depression and major depressive disorder among combined youth and adult populations in the U.S., Canada, U.K., Sweden, Netherlands, and others, report weights at 0.80 for the general population and ranging between 0.59–0.09 (for mild to severe depression) for patients without treatment and between 0.89–0.64 (for mild to severe depression) for patients with treatment (Sonntag et al., 2013).

Suicide prevention and intervention efforts need to be tailored to each target population to be relevant and effective. This is particularly true for AIAN youths and young adults, who have high rates of suicide and differ culturally and contextually from the broader U.S. population (and much more from the Australian, Dutch, or other nationals). Our study seeks to address the gap in the literature by sampling the general community of American Indian youth and young adults to derive culturally relevant utility weights for depression and suicide ideation. The Second Panel on cost-effectiveness in health and medicine recommends the use of community preferences together with a sensitivity analysis furnishing information on preferences of persons with the condition as a cross-check (Neumann et al., 2016). Likewise, our goal, in part, was to distinguish utility weights for suicide ideation as a distinct health state compared to depression for AIAN communities.

## Methods

### Data

We administered a health-related quality of life survey to AI youth and young adults ages 16–24. Previous research with the White Mountain Apache Tribe has indicated this age group is at particularly high risk for suicide (Mullany et al., 2009). The study included a questionnaire on demographic characteristics (i.e., age, gender, education, marital status, etc.) and a second survey eliciting participants’ self-reported rating of the quality of life (QoL) for suicide ideation and depression. The QoL measurement tool is an adaptation of the EuroQol’s visual analogue scale (VAS) (Herdman et al., 2011; The EuroQol Group, 1990). The VAS is a validated preference-based instrument widely used to measure utility weights (Szende et al., 2014; Szende & Williams, 2004).

### Study Population

The survey was administered between January and Feburary, 2020 to a street intercept sample of 200 eligible AI participants ages 16–24 who reside on or near the Fort Apache Indian Reservation, lands of the White Mountain Apache Tribe. The White Mountain Apache Tribe includes 17,500 tribal citizens and is a sovereign tribal nation governed by a Tribal Council including a Tribal Chair, Vice Chair, and 9 council members. One-third of the tribal population is between the ages of 10–24. In keeping with recommendations from the Second Panel on Cost-Effectiveness in Health and Medicine, the QoL measurements were drawn from a population without suicide ideation or depression in order to be most generalizable from a local community perspective (Neumann et al., 2016; Gillian D. Sanders et al., 2016a, 2016b; van Reenen & Janssen, 2015). Individuals who have or had a disease state under study have been found to systematically rate their health states higher (i.e., better) than the general population because of coping, adjustment and adaptation (Neumann et al., 2016; Sanders, & et al., 2016a, 2016b). As a validation check, a sample of youth experiencing suicide ideation but not depression and another of youth experiencing depression but not suicide ideation were also administered the survey (Neumann et al., 2016). Participants from both the general and patient populations were recruited through intercept recruitment at highly trafficked locations (e.g., grocery store, gas stations, after school center, and the gym). A subset of participants with suicide ideation or depressions was identified from the Celebrating Life suicide prevention program, which includes active community-based suicide surveillance and case management by a team of White Mountain Apache community mental health specialists. The Celebrating Life team also work as collaborative research partners on all studies, including the present study. They are trained in both protection of human subjects and followed Celebrating Life established guidelines to ensure safety of all participants (Mary F. Cwik et al., 2014). In the cases where potential individuals were identified as having a recent suicide attempt or suicide ideation (past 30 days), individuals, per tribal mandate, were reported to the suicide surveillance system and contacted by an assigned case manager.

Eligibility criteria for participation in the study included living on or near the Fort Apache Indian Reservation, age 16–24, and never attempted suicide. Those who reported that they ever attempted suicide or had suicide ideation in the past 3-months were referred to and followed-up by the celebrating life program to ensure participant safety. Assessment of depression symptoms was completed using the Center for Epidemiologic Studies Depression Scale (CESDR-10) survey (Andresen et al., 1994). Those with a CESDR-10 score of 8 or higher were considered at risk for clinical depression based on prior research and and thus eligible for the depression patient population study group, and those with a score lower than eight were eligible for the general population study group (Haroz et al., 2014). This score has been widely used among adolescent and AI populations, including Apache youth (Mary F Cwik et al., 2016; Radloff, 1977; Weissman et al., 1977). Participants who reported having suicide ideation in the past 3-months were only eligible for the patient sample with suicide ideation. All participants provided written informed consent and were given a $5 gift card as an honorarium for their time spent completing the demographic questionaire and study survey, which lasted approximately 5–10 min.

### Survey Design

There were important cultural adaptations related to designing and implementing the survey. First, the Apache study personnel identified that it would help if the vignettes were available both as an audio-recording and read by one of the study participants. They felt the audio-recording would overcome any issues with literacy and optimize the emotional and cognitive experience of the vignette. As such, the youth who recorded the vignettes had the same demographic characteristics as the non-patient study participants. A second cultural adaptation related to asking participants to rate the health of another person (i.e., Joe or Sarah) instead of their own. There are some cultural beliefs in Apache and other tribal traditions that discourage thinking or imagining bad things for oneself or others in direct proximity. Thus, the VAS component of the survey presented each participant with two different vignettes describing what it feels like for a hypothetical youth or young adult to have suicide ideation or depression. The vignette scripts were written and revised by White Mountain Apache community members, as well as community mental health specialists from the Johns Hopkins Center for American Indian Health, and then audio-recorded by AI voice actors. The vignettes and survey were presented to participants using tablet computers running the Qualtrics online survey platform.

After study consent, participants were handed study tablets with earphones to listen to the 2-min voice recordings of the vignettes. The text was also available to read. Gender-of-interviewer effects have been documented in video-web surveys and could be a source of bias (Fuchs, 2009). To minimize this bias and facilitate participants’ ability to identify with the vignettes, female participants heard the vignette read by a female voice that described a girl (Sarah) with either suicide ideation or depression; male participants heard a male voice that described the relevant health states for a boy (Joe). The order in which the two vignettes was presented was randomized for assurance against rating bias from offering another worse or less severe vignette before the second vignette. The vignettes and survey were piloted with 20 eligible study participants to ensure acceptability and feasability.

After listening to the recording, participants were immediately asked to rate the health of the individual described in the vignette on a horizontal visual analogue scale scored between zero and 100, where the endpoint zero was labeled ‘the worst health you can imagine’ and 100 was labeled ‘the best health you can imagine’ (van Reenen & Janssen, 2015). The question phrasing was: ‘We would like to know how good or bad you think Sarah’s/Joe’s health is TODAY. Please move the slider to indicate how you think Sarah’s/Joe’s health is TODAY.’ The VAS survey is designed for self-completion by respondents, and to be cognitively undemanding(van Reenen & Janssen, 2015). See the appendix for details about the vignettes and VAS instrument.

### Analysis

Descriptive statistics were used to describe participants’ demographic characteristics. Histograms and box-whiskers plots were used to assess the distribution of QoL data and whether values changed systematically with age. Given that data were skewed, the overall burden of suicide ideation and depression is reported with the median QoL values (van Reenen & Janssen, 2015). The Kruskal–Wallis test (a nonparametric statistical test) was used to evaluate if the distribution of QoL values for depression and QoL values for suicide ideation were equal in terms of their central tendency and dispersion. This test was repeated for subgroups stratified by key demographic characteristics (e.g., age, gender, marital status). Statistical analyses were undertaken using Stata version 14.2 (StataCorp, 2015).

## Results

Our study approached a total of 328 individuals to recruit 200 (61.0%) eligible and consenting participants for the general population sample (i.e., without suicide ideation or depression). Many of the general population participants were 18 years old or older (62%) and about half were female (51.0%). More than half of the sample reported being in school (55.0%): with the majority being in high school. For those not in school, the majority were unemployed (27.5%). See Table 1. We also recruited a small sample of 18 individuals with suicide ideation and 21 individuals with depression.

**Table 1:** Characteristics of the study population

Parameter	General population (n)	
Sample size	100.0%	(200)
Age category		
16 to 17	38.0%	(76)
18 to 24	62.0%	(124)
Gender		
Female	51.0%	(102)
Male	48.5%	(97)
Other	0.5%	(1)
Currently in school		
Yes	55.0%	(110)
No	45.0%	(90)
School grade/level		
Eighth	4.0%	(8)
Ninth	1.5%	(3)
Tenth	9.0%	(18)
Eleventh	20.0%	(40)
Twelfth	16.0%	(32)
GED program	0.5%	(1)
Associate’s/two-year program	4.0%	(8)
Not in School	45.0%	(90)
Highest degree or level of education today		
Less than high school	3.0%	(6)
Some high school	46.0%	(92)
A high school/GED diploma	37.5%	(75)
Some college (less than 2 yrs.)	8.5%	(17)
An associate’s degree	1.0%	(2)
Some college (2 or more yrs.)	2.0%	(4)
A bachelor’s degree	2.0%	(4)
Main current activity		
Student	53.0%	(106)
Employed/self-employed	16.5%	(33)
Unemployed	27.5%	(55)
Other	3.0%	(6)
Marital status		
Single (never married)	93.5%	(187)
Married (living w. partner)	6.0%	(12)
Widowed	0.5%	(1)

**Fig. 1:**
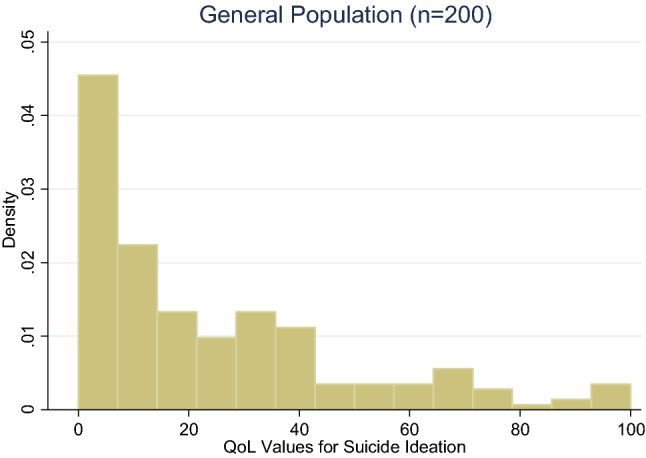
Distribution of QoL values for suicidal ideation. The scale of QoL values is 0–100 where 0 is worst health and 100 is best health

**Fig. 2:**
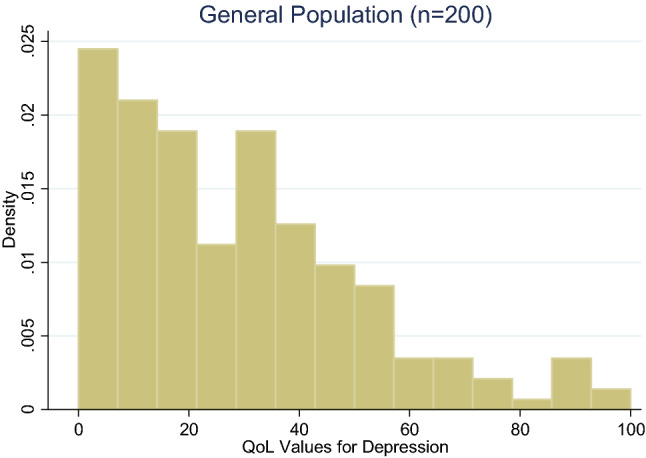
Distribution of QoL values QoL for depression. The scale of QoL values is 0–100 where 0 is worst health and 100 is best health

The distribution of QoL values among the general population was skewed to the right for suicide ideation and depression scores; see Figs. 1 and 2, respectively. For the general population, the median utility wights (i.e., “quality of life rating” or QoL) for suicide ideation was 15.8 (25th and 75th quartiles were 2.8 and 36.3, respectively) and for depression was 25.1 (25th and 75th quartiles were 10.3 and 40.6, respectively), see Table 2, respectively. The same tables list QoL values by age group, and the appendix Figure A1-2 show the distribution of QoL by health outcome and age. For both health outcomes, QoL seems to rise until age 21 and then fall, but it does not monotonically increase or decrease the QoL response. The QoL values for the subgroup of participants currently experiencing suicide ideation or depression were slightly higher than those of the general population, 19.3 and 28.0 for suicide ideation and depression, respectively.

**Table 2:** Quality of life ratings for suicide ideation and depression

Age	Suicide Ideation						Depression					
	Median	25th	75th	Min	Man	N	Median	25th	75th	Min	Man	N
	General population						General population					
16	10.9	4.4	26.8	0.0	100.0	45.0	15.5	7.6	40.6	0.0	100.0	45.0
17	20.5	2.2	36.1	0.0	89.3	27.0	30.0	11.0	50.3	0.0	91.9	27.0
18	20.4	7.1	38.8	0.0	95.0	32.0	21.0	10.5	31.3	0.0	65.8	32.0
19	11.0	2.0	40.8	0.0	71.0	25.0	25.1	17.6	34.4	0.0	65.8	25.0
20	23.9	7.0	44.8	0.0	100.0	16.0	27.9	18.1	40.2	9.9	62.9	16.0
21	24.0	10.1	41.7	0.0	100.0	17.0	41.1	27.6	50.3	0.0	100.0	17.0
22	8.1	0.0	21.6	0.0	64.5	17.0	21.1	9.0	40.2	0.0	50.3	17.0
23	15.9	3.7	31.1	0.0	59.9	12.0	18.3	10.3	42.9	0.0	73.7	12.0
24	9.3	6.0	14.7	0.0	40.7	5.0	30.7	27.7	39.4	9.6	39.9	5.0
**All**	**15.8**	**2.8**	**36.3**	**0.0**	**100.0**	**200.0**	**25.1**	**10.3**	**40.6**	**0.0**	**100.0**	**200.0**
	Patient population with suicide ideation						Patient population with depression					
All	19.3	0	38.8	0	86	18	28	17	40.1	0	100	21

The row in bold shows the overall result for the general population

Table 3 lists QoL median values and the Kruskal–Wallis test results for the overall study population and subgroups stratified by demographic characteristics. Overall, there was a statistically significant difference between the sample of QoL statements about suicide ideation and statements about depression, where the former is significantly worse than the latter, and this difference holds in all demographic characteristic subgroups (p < 0.05), except for the 55 respondents that were unemployed (p-value was not < 0.1).

**Table 3:** Difference in the QoL median by health outcome and demographic characteristic

Demographic characteristic	Groups ±	Obs	QoL median		Equality test p-value^†^
			Suicide ideation	Depression	
All		200	15.80	25.10	***
Age group	16 to 17	76	12.10	23.25	**
	18 to 24	124	19.05	26.60	**
Gender	Female	102	12.05	20.85	**
	Male	97	19.70	30.00	**
Current main activity	Student	106	17.30	25.15	**
	Employed	33	12.10	27.70	**
	Unemployed	55	18.40	23.70	
Marital status	Single	187	15.80	25.10	**
	Married	12	15.85	18.80	**

± Groups with 10 or more observations (obs.). ^†^Equality of sample distribution (Kruskal–Wallis test). ***p < 0.01, **p < 0.05, *p < 0.10, no asterisk means p-value is greater than 0.09. p-values less than 0.05 mean that the sample of quality of life (QoL) statements about suicide ideation and statements about depression are not from the same population (i.e., there is a statistically significant difference in the median QoL). Scale of QoL values is 0–100 where 0 is worst health and 100 is best health. We also used a Kruskal–Wallis test to check whether QoL scores were statistically significantly different across age group, gender, main activity, and marital status and found statistically significant differences for age group, gender, and economic activity

## Discussion

We present the first estimates of quality of life for the states of suicide ideation and depression within one community sample of American Indian youth and young adults from a Southwest reservation. Our results indicate that among the study population, the utility weights (i.e., “quality of life values” or QoL) for suicide ideation and depression are 15.8 and 25.1, respectively. On a scale between 0 and 100, where zero is the worst imaginable health and 100 is the best imaginable health, the general community rates both of these conditions as quite low (or bad), and as expected, living with suicide ideation is rated worse than living with depression. The QoL values for suicide ideation and depression were also statistically significantly different for subgroups of different sociodemographic characteristics, except for the subgroup of youth who were unemployed, and suicide ideation was consistently rated lower than depression across subgroups. However, the study sample size was not powered to capture differences for subgroups. Similarly, as expected, the cross-check comparing the QoL values between a sample of individuals experiencing suicide ideation or depression and the general population (19.3 vs. 15.8 and 28.0 vs. 25.1, respectively), confirmed that the currently symptomatic populations rated QoL in these states higher (i.e., not as bad) than the general population (Neumann et al., 2016).

Comparing results to other studies is not feasible because utility weights are unique to the population context, and this is the first study valuing health states for AIAN youth. However, relative to other cultures, based on two studies using the same instrument, the utility weight for suicide ideation for this study population (15.8) is substantially lower (or worse) than weights for the general population of Australia (45) and a sample of medical practitioners in the Netherlands (36) (van Spijker et al., 2011). Similarly, depression utility weights (25.1) for this study population are substantially lower compared to the utility weights for the general population (80) and youth patient population without treatment (57) in the U.K., U.S., and Netherlands (Sonntag et al., 2013). Overall, the low value of the utility weight for depression and suicide ideation among the youth in the AI community shows greater perceived severity compared to older age groups afflicted with the same health status. Note some of the values reported from prior literature were transformed from the 0–1 scale to a 0–100 scale for comparison purposes.

The high disability burden for youth and young adults experiencing these health conditions in AIAN communities, particularly suicide ideation, is a significant public health concern. This concern is further heightened by the substantial mortality burden from suicide in the U.S. (i.e., 17.3% of all deaths among the 10–24 year olds in the U.S.). Further, among AIAN youth, suicide accounts for 26.0–29.4% of all deaths, depending on the age group and tribe (Heron, 2018). The high disability and mortality burden from suicide ideation makes it urgent to develop and expand cost-effective interventions that can promptly identify, treat, and prevent the onset of these illnesses.

It is important to note that the cultural adaptations to the methods for estimating QoL in this population—including audio-recording vignettes so participants could listen to the stories, in addition to rating the health state for a “character” rather than for oneself—may offer innovations to primary data collection in this field. However, further research is needed to understand if these methods can elicit more reliable responses in this and other populations.

### Limitations

There are a few limitations to this study. First, the selection of participants with or without suicide ideation or depression was based on participants’ self-report to a direct question and the CESDR-10 score, respectively. Self-reported data may result in bias if participants believe that higher or lower scoring is socially desirable or self-comforting. We have limited means of identifying the direction or size of the bias at this time. However, given that the sample of the general population had slightly lower estimates than the symptomatic sample and that both groups rated these conditions as having very low QoL, it is clear that these health states are of priority concern to the population sub-group who was surveyed—or are the highest risk age-group for suicide.

Likewise, our study sample was drawn from groups of youth using local intercept recruitment and sample selection might make our participants unlike a representative sample of schools or households. We chose to recruit participants from high-traffic public areas to reduce recruitment costs. Recruiters did not specifically target eligible subjects on their appearance or affect except to try to guess their age group. Participants could opt out or opt into consent after learning that the survey was about mental health states and this may have altered their decision to enroll, but household-based recruitment would face the same potential sample selection bias. The study sample included participants that reside on or near the Fort Apache Indian reservation, and they may not be representative of tribe members living in other settings or of other tribes. Future research should replicate this study on wider populations given the vast diversity among 574 federally recognized tribes and 100+ state recognized tribes located in diverse regions and differences found among those who live on reservations (22%) versus those in urban areas (78%) in the U.S. (National Congress of American Indians, 2020; Norris et al., 2012; Office of Minority Health Resource Center, 2018).

Lastly, the VAS instrument has been viewed as a less favorable option for eliciting utility values in economic evaluations compared to the EQ-5D instrument (Sonntag et al., 2013; Stiggelbout et al., 1996). The EQ-5D is an indirect valuation method based on a generic multi-attribute questionnaire (The EuroQol Group, 1990). Valuation of health status with the EQ-5D requires a generic value set of predefined health states (The EuroQol Group, 2020). This value set is available for the general U.S. population (Pickard et al., 2019) but not for AIAN communities. The difference in the valuation performance between these instruments may arise from both context and end-aversion bias related to respondents’ cognitive processes (Torrance et al., 2001). The former can depress or enhance the health valuation if many better or worse states are presented beforehand. The latter refers to the reluctance of some respondents to use extreme categories. While it would not have been possible to use the EQ-5D in our study, we follow the main recommendations to mitigate bias by including the valuation of only two health states, randomly changing the order in which each case study was presented to each participant, and adjusting and piloting the description and labeling of text and scale points ensuring clarity and cultural appropriateness. The VAS survey was advantageous in this setting because it is simple, quick to administer, and lends itself to self-completion. Despite these limitations, this study’s inclusion of extensive input from the study population and administration by a data collection team with a strong trusting relationship with the community offered important strengths.

## Conclusion

A general population of 200 AI youth and young adults were presented with vignettes describing states of suicide ideation and depression and rated these conditions as having low QoL scores at 15.8 and 25.1, respectively, indicating a diminished quality of life. As expected, the general population’s ratings were slightly lower than validation subsets of 18 youth currently symptomatic with suicide ideation and 21 youth with depression who rated these states with QoL of 19.3 and 28.0, respectively. Our findings also help to distinguish suicide ideation as a distinctly worse health state than depression for AI communities. The QoL weights for youth and young adults are very low indicating high disability burden. Results suggest the importance of identifying effective interventions that reduce suicide ideation as a community priority and to prevent those with depression symptoms from experiencing suicide ideation. Weight estimates from this study will improve the ability to evaluate the cost-effectiveness of interventions targeting reduced depression and suicide. Similarly, culturally specific QoL values for AIAN youth and young adults will allow identification and comparison of the subset of effective and feasible programs and policies appropriate for this specific population. Evidence from such studies can guide IHS and tribally run health departments, healthcare providers, and tribal nations in decisions about resource allocation and adoption or expansion of programs that best mitigate onset of suicide ideation. Prevention and recovery from depression and/or suicide ideation, as well as maintaining reasons for living remain the ultimate goals.

## Supplementary Information

Below is the link to the electronic supplementary material.

Supplementary file1 (PDF 203 kb)

## Data Availability

The White Mountain Apache Tribe has exercised sovereignty over their data and opted to retain stewardship over it, as such it is not possible to share the data. However, the analysis code, output log, and an explanatory memo of these files is available upon request to provide details about the analysis. Any questions readers may have about data may be extended to the authors for further explanation.
