# Supplementary material for: Suicide Ideation and Depression Quality of Life Ratings in a Reservation-Based Community of Native American Youths and Young Adults
Source: Community Ment Health J. 2021 Aug 29;58(4):779–87. doi: 10.1007/s10597-021-00883-w (PMC8933312; doi:10.1007/s10597-021-00883-w)

Suicide ideation and depression quality of life ratings in a reservation-based community of Native American Youths and Young Adults

Supplemental Material
Appendix

Contents

VIGNETTES 1

Suicide Ideation 1

Depression 1

SURVEY DESIGN 2

Appendix Figure 1. VAS survey question 2

RESULTS 3

Box-Whisker Plots of QoL and Age 3

Appendix Figure A1. Median and dispersion of QoL values for Suicide Ideation by Age 3

Appendix Figure A2. Median and dispersion of QoL values for Depression by Age 3

VIGNETTES

For female participants, the vignette for suicide ideation talked about a girl with the name Sarah and for depression with the name Emily. For all other participants, the vignette for suicide ideation talked about a boy with the "name" Joe and for depression with the name Michael. These are names common among youth and young adults in the Indian American community.

Suicide Ideation

"[Name] has been thinking that life is not worth living, especially when he is alone. He feels like no one really loves him and that his friends and family would be happier if he were not around. He thinks a lot about death and dying, and has even thought about how he would end his life. Sometimes he feels like suicide is the only option – that it would solve all his problems. [Name] is having trouble getting these negative thoughts to stop, they're like a broken record playing over and over. When he has trouble at school, trouble finding a job, and has a disagreement with his family or friends, he thinks that no one would care if he died. He feels guilty and even shame and sometimes has given his things away and said goodbye to friends and family as if it were the last time he would see them. He has had these thoughts on and off for the past month."

Depression

"For the past several weeks, [Name] has been feeling sad almost every day. He has lost interest in things he usually enjoys like playing basketball and hanging out with friends. [Name] does not want to talk about his feelings with anyone, avoids people and wants to be left alone. He doesn't eat very much during the day, and he often stays up until late at night because he can't sleep. [Name] is tired most of the day, skips meals and has trouble concentrating. [Name]'s self-confidence is very low since he stopped playing basketball and he is struggling at school, but he doesn't feel like he has the energy to do much of anything."

SURVEY DESIGN

Appendix Figure 1. VAS survey question

VALUING EMILY'S HEALTH TODAY

The following section will present a case study about the health status of a girl named Emily. The case study will be described to you using both a voice recording and a short text. You will be able to hear and or read the text of what she says. After the case study, you will be asked to rate Emily's health state. There are two case studies in total.

CASE STUDY

Please CLICK PLAY to listen to the audio describing Emily's health. You can also read the description below.

0:00 / 0:37

"For the past several weeks, Emily has been feeling sad almost every day. She has lost interest in things she usually enjoys like playing basketball and hanging out with friends. Emily does not want to talk about her feelings with anyone, avoids people and wants to be left alone. She doesn't eat very much during the day, and she often stays up until late at night unable to sleep. Emily is tired most of the day, skips meals and has trouble concentrating. Emily's self-confidence is very low since she stopped playing basketball and she is struggling at school, but she doesn't feel like she has the energy to do much of anything. "

We would like to know how good or bad you think Emily's health is TODAY.

This scale is numbered from 0 to 100:

100 means the **best health** you can imagine for Emily.

0 means the **worst health** you can imagine for Emily.

Please **move the slider** to indicate **how you think Emily's health is TODAY**.

0 is the **worst** 100 is the **best**

0 10 20 30 40 50 60 70 80 90 100

RESULTS

Box-Whisker Plots of QoL and Age

Appendix Figure A1. Median and dispersion of QoL values for Suicide Ideation by Age

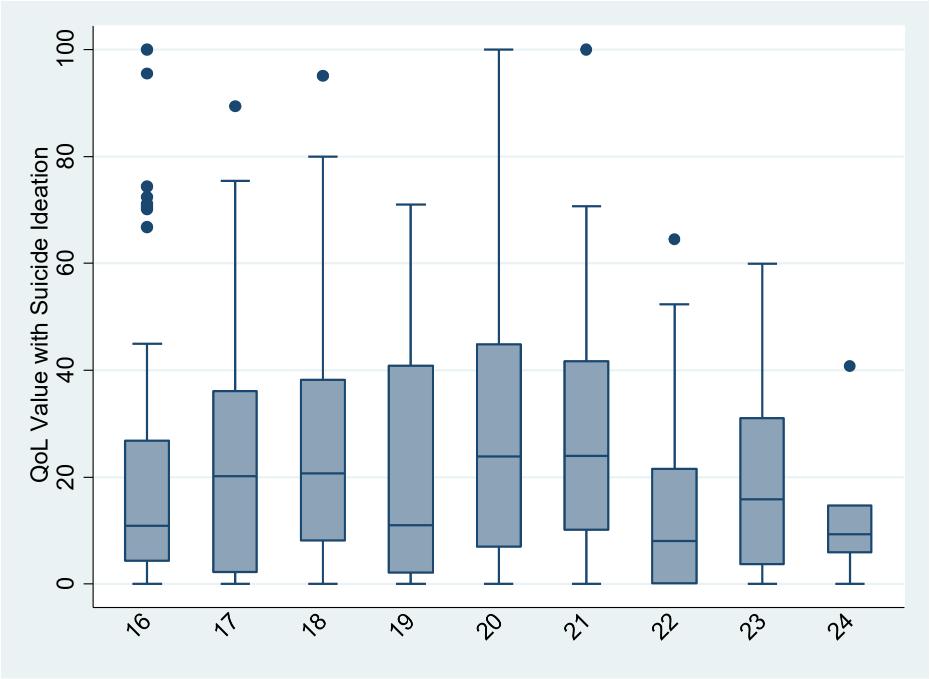

Appendix Figure A2. Median and dispersion of QoL values for Depression by Age

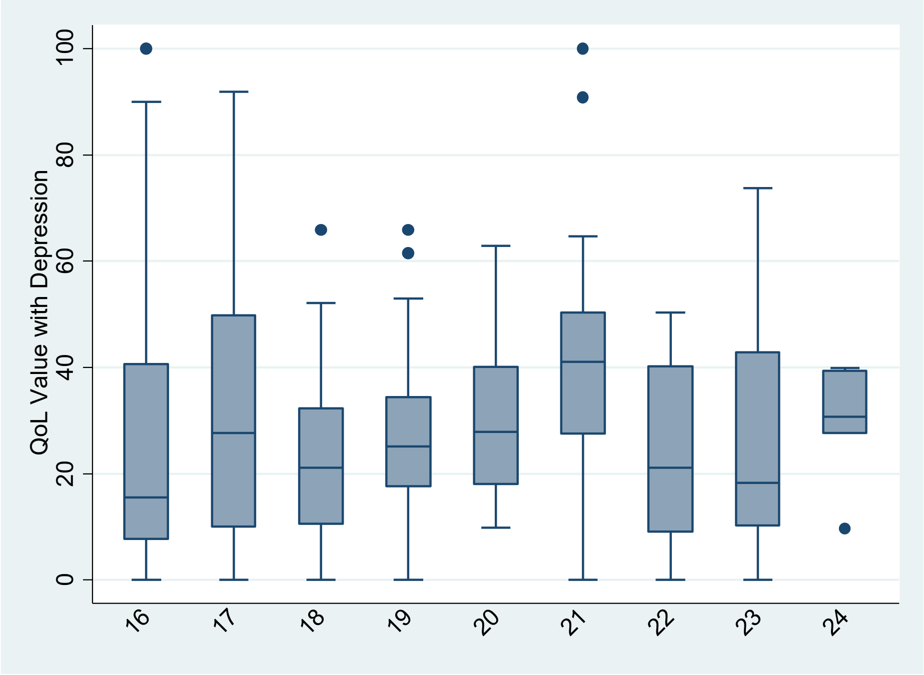

Supplement: Supplementary file 1 — Supplementary file1 (PDF 203 kb) [file 10597_2021_883_MOESM1_ESM.pdf]
